# Ultra-wideband, Wide Angle and Polarization-insensitive Specular Reflection Reduction by Metasurface based on Parameter-adjustable Meta-Atoms

**DOI:** 10.1038/srep42283

**Published:** 2017-02-09

**Authors:** Jianxun Su, Yao Lu, Hui Zhang, Zengrui Li, Yaoqing (Lamar) Yang, Yongxing Che, Kainan Qi

**Affiliations:** 1 College of Information Engineering, Communication University of China, Beijing 100024, China; 2 Department of Electrical and Computer Engineering, University of Nebraska-Lincoln, NE, 68182, USA; 3 Science and Technology on Electromagnetic Scattering Laboratory, Beijing, 100854, China

## Abstract

In this paper, an ultra-wideband, wide angle and polarization-insensitive metasurface is designed, fabricated, and characterized for suppressing the specular electromagnetic wave reflection or backward radar cross section (RCS). Square ring structure is chosen as the basic meta-atoms. A new physical mechanism based on size adjustment of the basic meta-atoms is proposed for ultra-wideband manipulation of electromagnetic (EM) waves. Based on hybrid array pattern synthesis (APS) and particle swarm optimization (PSO) algorithm, the selection and distribution of the basic meta-atoms are optimized simultaneously to obtain the ultra-wideband diffusion scattering patterns. The metasurface can achieve an excellent RCS reduction in an ultra-wide frequency range under x- and y-polarized normal incidences. The new proposed mechanism greatly extends the bandwidth of RCS reduction. The simulation and experiment results show the metasurface can achieve ultra-wideband and polarization-insensitive specular reflection reduction for both normal and wide-angle incidences. The proposed methodology opens up a new route for realizing ultra-wideband diffusion scattering of EM wave, which is important for stealth and other microwave applications in the future.

Metasurface, a new class of metamaterials that consist of a monolayer of planar metallic structures, is capable of generating abrupt interfacial phase changes and providing a unique way of fully controlling the local wave front at the subwavelength scale. A plethora of applications have already been proposed and demonstrated by using metasurfaces such as wave plates for generating vortex beams^1^,^2^, ultrathin metalenses^3^,^4^, polarization converter^5^, polarization and frequency reconfiguration^6^,^7^, and electromagnetic interference and shielding^8^,^9^. One of the potential applications of metasurface is to reduce the specular EM wave reflection or backward RCS of an object, which is of great significance in military field.

Generally, the RCS reduction can be achieved by two ways. One is electromagnetic wave absorption, and the other is phase cancellation or destructive interference. For the absorptive method, the radar absorbing metamaterial/metasurface can also be used for RCS reduction by transforming electromagnetic energy into heat^10^,^11^,^12^,^13^,^14^. However, radar absorbing metamaterials usually operate in the vicinity of resonance frequency. For the phase cancellation method, the scattered energy could be redirected away from the source direction. The basic idea is to exploit the cancellation effects arising from the well-known 180° phase difference between the corresponding reflection coefficients. In Paquay *et al*.^15^ proposed a planar structure for RCS reduction, based on a combination of artificial magnetic conductors (AMC) and perfect electric conductors (PEC) in a chessboard-like configuration^15^. The backscattering field can be effectively canceled by redirecting it along other angles. However, the narrow in-phase reflection bandwidth of the AMC restricts the RCS reduction frequency range. In ref. 16, a planar monolayer chessboard structure is presented for broadband radar cross-section reduction by using AMC technology. More than 40% frequency bandwidth with a monostatic RCS reduction larger than 10 dB is obtained. In ref. 17, nonabsorptive two-layered miniaturized-element frequency selective surfaces of a chessboard-like configuration have been proposed for wideband RCS reduction. The −6 dB monostatic and bistatic RCS reduction bandwidth of 66% is achieved at the normal incidence. In another similar work^18^, chessboard AMC surface is proposed to reduce the monostatic RCS of microstrip antenna with an electrically large ground. These rectangular checkerboard surfaces^15^,^16^,^17^,^18^ of periodic phase arrangement create four scattering beams and bistatic RCS reduction is about 8.1 dB. In W. Chen and C. A. Balanis *et al*. proposed a hexagonal checkerboard surface^19^ of periodic phase arrangement, with the −10 dB monostatic RCS reduction bandwidth of about 61%, which can create six bistatic RCS lobes, leading to further bistatic RCS reduction by 8.9 dB.

More recently, coding or digital metamaterial/metasurface has been proposed for wideband RCS reduction^20^,^21^,^22^,^23^,^24^,^25^,^26^,^27^,^28^. The design process is divided into two independent steps. The first step is to select meta-atoms, the bandwidth of phase difference between them to determine the bandwidth of RCS reduction. The second step is to find the phase layout, which determine the scattering pattern. To generate diffusion effect, pseudo-random codes^22^,^23^,^24^,^25^ or certain optimization algorithms together with array pattern synthesis^26^,^27^,^28^ are utilized to achieve the layout of the meta-atoms. The RCS reduction exploits the destructive interference arising from these meta-atoms. For an *N*-bit coding metamaterial, it is very difficult to design 2^*N*^ meta-atoms with the fixed phase difference of 360°/2^*N*^ in a broad frequency band. Currently, 3-bit coding metamaterials can be achieved. In ref. 27, T. J. Cui *et al*. proposed 3-bit coding metasurfaces for broadband diffusion of terahertz waves. The bandwidth of backward scattering coefficients less than 0.2 is about 77%. In ref. 25, a 3-bit coding metasurface based on multi-resonant polarization conversion elements is proposed. The bandwidth of −10 dB RCS reduction is extended to 89.9% (7.9–20.8 GHz) under normal incidence. However, the performance of polarization conversion element is sensitive to incident angle. In microwave application, development of a new approach to realize ultra-wideband, wide angle and polarization-insensitive specular reflection reduction is highly desirable and challenging.

In this paper, a new mechanism based on geometric parameter optimization is proposed for realizing ultra-wideband manipulation of EM waves. By adjusting the size of basic meta-atoms, we can achieve different reflection phase distributions for the metasurface in ultra-wide frequency band. The selection and arrangement of the basic meta-atoms are carried out simultaneously to achieve ultra-wideband diffusion EM wave scattering by hybrid array pattern synthesis (APS) and particle swarm optimization (PSO) algorithm. The metasurface can achieve ultra-wideband RCS reduction from 11.3 to 51.3 GHz with the reflection coefficients less than 0.2 for normal x- and y-polarized incident waves. For wide angle incidence of arbitrary direction and polarization, the proposed metasurface still performs well in the operating frequency band. The simulated and measured results validate the effectiveness of the newly proposed mechanism for greatly extending the bandwidth of RCS reduction. Therefore, the proposed design opens up a new route for ultra-wideband suppression of specular reflection, which is important for stealth and other microwave applications in the future. The mechanism of the proposed metasurface mainly depends on the geometric parameters and distribution of the basic meta-atoms, which is totally different from the mechanisms of previous researches.

## Results

### Unit cell design

**Figure 1:**
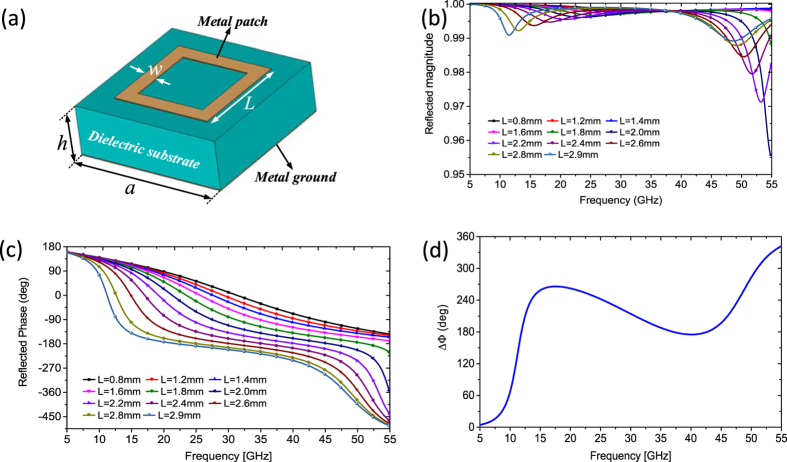
Geometry of the basic meta-atom and its reflection properties. (**a**) Geometry of square ring element. Dimensions are: *a* = 3, *w* = 0.3, *h* = 1.5748 in mm. (**b**) Reflection magnitude and (**c**) Reflection phase versus frequency with the change of side length *L*. (**d**) The available phase coverage in the frequency range from 5 to 55 GHz. The subtraction is made between two phase curves of the smallest size (*L* = 0.8 *mm*) and biggest size (*L* = 2.9 *mm*).

Square ring patch was chosen as the basic meta-atom of the metasurface for its reflection phase characteristics, simplicity of design, and ease of fabrication. When the frequency is fixed, the reflection phase range with the change of side length is relatively large. The unit cells are printed on the surface of Rogers RT/duroid 5880 substrate with a thickness of 1.5748 mm and a dielectric constant *ε*_*r*_ = 2.2 (loss tangent tan *δ* = 0.0009). The geometry of the meta-atom is illustrated in Fig. 1(a). The meta-atoms are simulated by Ansoft Designer based on Method of Moments (MoM). The periodic boundary condition (PBC) is imposed on unit cells to create infinite structure and obtain their reflection magnitude and phase. In this simulation, side length *L* of the meta-atoms varies from 0.8 to 2.9 mm with a step size of 0.01 mm while other dimensions such as the periodicity *a* of unit cell, the width *w* of square ring and the thickness *h* of dielectric substrate, are fixed. A part of reflection coefficient curves in the 5–55 GHz range are plotted in Fig. 1(b and c). The magnitude of the reflection coefficient is unity due to the infinite metallic ground plane, while the available phase coverage by tuning the side length of meta-atom is larger than 176° (see Fig. 1(d)) at frequencies range from 11 to 55 GHz. This phase feature guarantees the possibility of ultra-wideband manipulation of EM waves.

**Figure 2:**
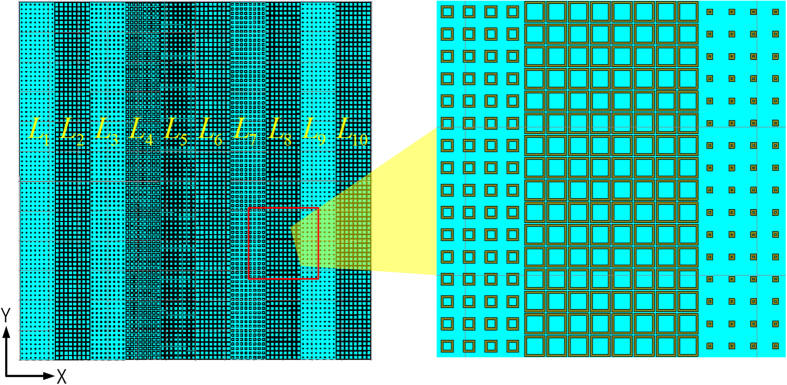
Geometry of proposed ultra-wideband metasurface. It consist of ten lattice strips in which the size (*L*_1_, *L*_2_, … *L*_10_) of basic meta-atoms can be arbitrary values between 0.8 mm and 2.9 mm.

To satisfy the periodic boundary condition (PBC) used in simulation, each lattice is occupied by a subarray of 8 × 8 unit cells. The metasurface contains 10 × 10 lattices. For simplicity, a 1-D metasurface is studied in this paper to verify the proposed physical mechanism for ultra-wideband manipulation of EM waves, as shown in Fig. 2. For the lattices along y-direction, the sizes of the meta-atoms are identical. Thus, the metasurface consists of 10 lattice strips in which the size (*L*_1_, *L*_2_, … *L*_10_) of the meta-atoms can be adjustable along x-direction.

### Optimal Design of Metasurface

Each lattice strip is considered to be an independent array element with a particular phase and array theory is applied to analyze the scattering features of the metasurface. The 1-D metasurface shown in Fig. 2 can be considered as a 10-element linear array. Due to that the reflection magnitude of the basic meta-atoms is unity, the far field scattering pattern is predicted using phase-only array pattern synthesis (APS).

**Figure 3:**
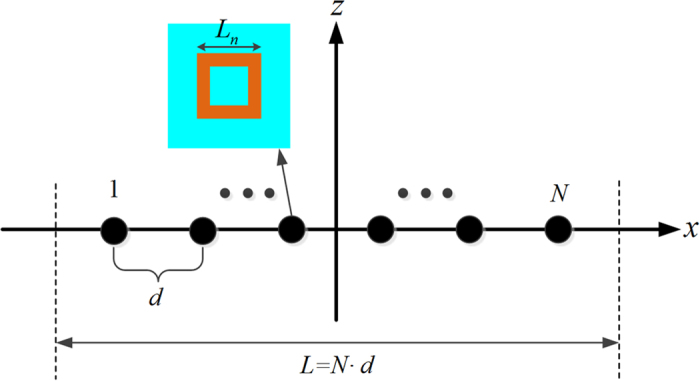
Linear array of *N* elements. The phase of each array element is the reflection phase of lattice strip, and is determined by the side length *L*_*n*_ of the meta-atoms. The magnitude of each array element is unity because of the infinite metal ground.

For an *N*-element linear array with uniform space of the lattice width *d* depicted in Fig. 3, the far field scattering pattern of the metasurface at angle *θ* can be expressed as^29^


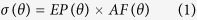


and


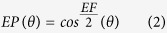



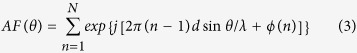


where *EP* and *AF* are the element pattern and the array factor, respectively. A cosine function, raised to a power that called the element factor (*EF*), is used to model the normalized element pattern. *θ* is the elevation angle of an arbitrary scattering direction, and *ϕ(n*) is the reflection phase of the *n-th* basic meta-atom with side length *L*_*n*_. By introducing a new variable *U* = *sinθ* in Eq. (3), we have *AF(U* + *λ*/*d*) = *AF(U*). Thus, array factor *AF(U*) is a periodic function with the period *p* = *λ*/*d*. Due to the attenuation characteristic of *EP* as the angle *θ* increases, the maximum of bistatic RCS appears in the main period where *U* is from −*λ*/2*d* to *λ*/2*d*. Thus, only far field scattering pattern in the main period is considered, while scattered fields away from the main period are weakened by the *EP*. Note that Eq. (1) can provide the predicted RCS pattern of the metasurface if Eq. (2) uses the simulated RCS pattern of element pattern.

Reflection phase of the basic meta-atom at a specific frequency is determined by its shape and size. In this paper, geometric parameter optimization is based on size adjustment, while the profile (square ring) keeps the same. Tuning the size of basic meta-atoms can change reflection phase distribution of the metasurface in a frequency band, resulting in the change of far field scattering patterns at this frequency band. To achieve the best performance of the metasurface, particle swarm optimization (PSO) algorithm is employed to obtain the optimal sizes and distribution of ten basic meta-atoms due to its merits of simple description and high efficiency. The main objective of this study is to produce a flat scattering pattern with the lowest amplitude of the main lobe in an ultra-wide frequency band. In optimization, the scattering patterns of a sequence of optimization frequencies sampled in an ultra-wide frequency band are considered. The fitness function is therefore defined as


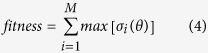


where *σ*_*i*_ is the bistatic RCS pattern at *i-th* frequency point. *M* is the number of optimization frequencies.

**Figure 4:**
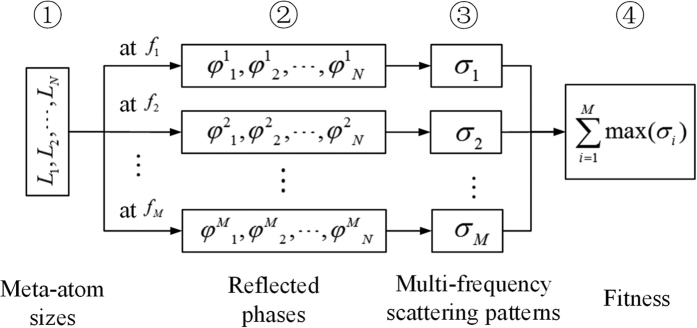
Schematic diagram for ultra-wideband manipulation of EM wave by geometric parameter adjustment.

The schematic diagram for ultra-wideband manipulation of EM waves through geometric parameter adjustment is illustrated in Fig. 4. It also describes the scoring process for PSO in detail. *N* is the number of basic meta-atoms of the metasurface, which equals 10 in this design. The reflected phase versus frequency with the changes of side length *L* simulated in Fig. 1(c) are pre-stored in a table. For a combination of the basic meta-atoms, *M* combinations of reflected phases at the optimization frequencies can be read from the phase table. These phase combinations can result in *M* scattering patterns. Finally, these *M* scattering patterns can be used to evaluate the corresponding fitness. Since the changes of meta-atom size in simulation are discrete, polynomial curve fitting method is used to create the features of the continuous reflected phase versus side length *L* at the optimization frequencies.

**Figure 5:**
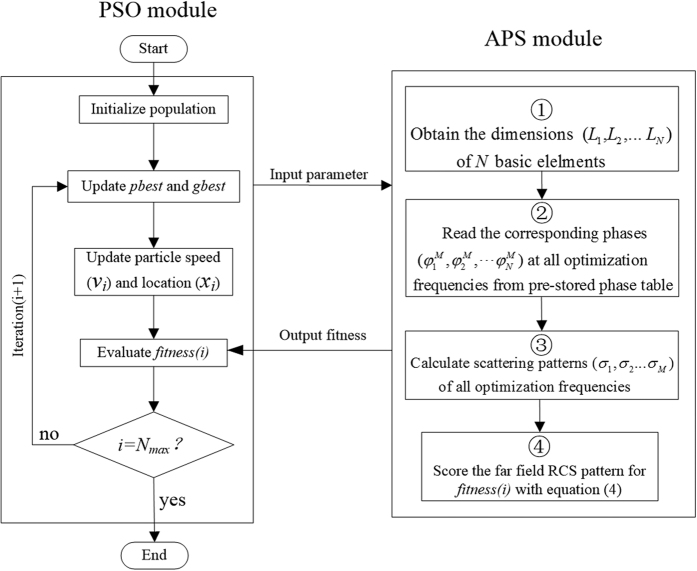
The code flowchart for the optimal design of ultra-wideband low-RCS metasurface.

The flow-chart of hybrid optimization algorithm for designing the ultra-wideband low RCS metasurface is shown in Fig. 5, where two modules have been used to achieve the desired optimal design. The array pattern synthesis (APS) module based on Eq. (1) calculates the scattering patterns of metasurface and provides the PSO module with numerical basis for evaluating the fitness of the size combination. The PSO module updates the sizes of the ten meta-atoms, and evaluates the fitness of these various size combinations. In PSO algorithm, each particle is treated as an individual in the population who searches for the optimum solution through individual cooperation and information sharing^30^,^31^. Each particle keeps track of its coordinates (x) which are associated with the individual best solution (*gbest*) and the global best solution (*gbest*) that has been achieved. The fitness value of potential solution is scored by an appropriate fitness function. The mathematical description of the speed and location of *i-th* particle of the next generation ( *j* + 1) can be expressed as^32^


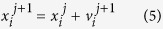


and


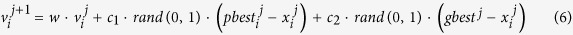


where 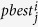 is the individual optimal location and *gbest*^*i*^ is the global optimal location. The function *rand*(0, 1) indicates a random number chosen from a uniform distribution between 0 and 1, *c*_1_ and *c*_2_ are two acceleration constants that are usually set to be 2.0 from past experience^32^,^33^,^34^, and *w* is the inertia weight factor of 0.4. When the loop reaches the maximum number of iterations (*N*_*max*_), the optimization process ends.

**Figure 6:**
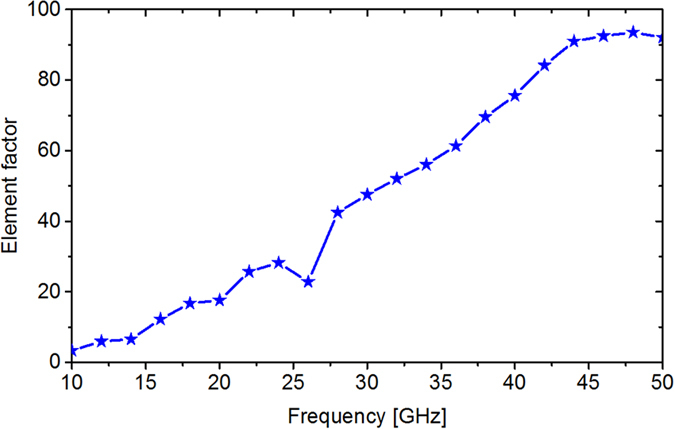
Element factor (*EF*) of the cosine function used to fit the normalized element pattern (*EP*). Twenty one scattering patterns for the lattice strip from 10–50 GHz with an interval of 2 GHz are simulated and fitted by cosine function. As frequency increases, the beam width of element pattern becomes smaller and element factor becomes larger. The *EF* values at optimization frequencies are obtained by polynomial curve fitting method.

**Figure 7:**
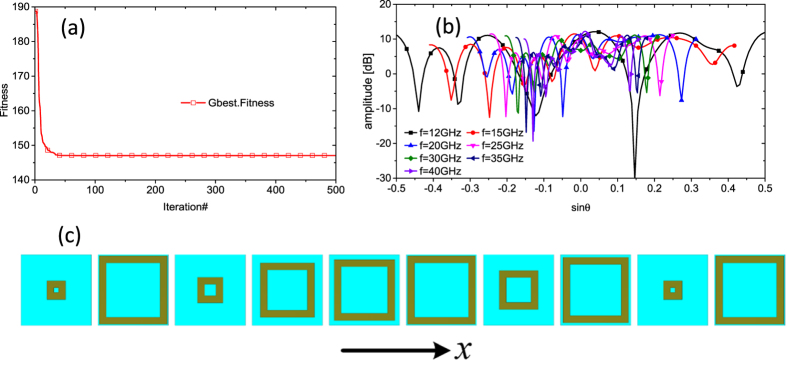
The particle swarm optimization for finding the optimal dimensions and distribution of ten basic meta-atoms. (**a**) The evolution plot of fitness. (**b**) The predicted scattering patterns in the main period of a part of optimization frequencies. (**c**) Ten optimal meta-atoms and its distribution. The side lengths (*L*_1_, *L*_2_, … *L*_10_) are 0.8 mm, 2.9 mm, 1.0811 mm, 2.3059 mm, 2.5833 mm, 2.9 mm, 1.6558 mm, 2.7846 mm, 0.8 mm and 2.9 mm in sequence.

In this optimization process, a total of 40 optimization frequencies from 11 to 50 GHz with an interval of 1 GHz were considered. A 100-agent swarm for 500 iterations is used in PSO module. The normalized scattering pattern of the lattice strip is exactly fitted by Eq. (2), and the corresponding *EF* value curve versus frequency is plotted in Fig. 6. Initial size of the basic meta-atom is a random number chosen from a uniform distribution between 0.8 mm and 2.9 mm. When 500 iterations finished, we can get the optimal dimensions and distribution of ten basic meta-atoms for the metasurface with lowest RCS in a desired ultra-wide frequency band. The total processing time for optimization is 83 s. The evolution plot of *fitness* is given in Fig. 7(a). It can be seen that the curve decreases rapidly during the inception phase and then it tends to stabilize, showing the high efficiency of the hybrid optimization algorithm. A part of its corresponding scattering patterns in the main period is depicted in Fig. 7(b). It is shown that the scattered field can be redirected to more directions at all optimization frequencies. Approximate diffusion reflections are generated in an ultra-wide frequency band. As a result, the maximum value of the scattered field can be reduced due to energy conversation, leading to an ultra-wideband RCS reduction. The optimal sizes and distribution of ten basic meta-atoms are depicted in Fig. 7(c). The advantage of our approach is that selection and arrangement of basic meta-atoms are carried out simultaneously by the hybrid optimization algorithm to realize ultra-wideband diffusion of EM wave. For different frequencies, the phase layout response of the metasurface is different, resulting in different diffusion scattering pattern.

### Simulation and Measurement

**Figure 8:**
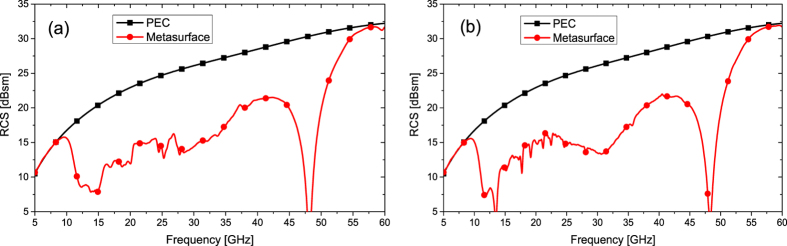
The simulated RCSs for the proposed metasurface and the equal-sized PEC surface. (**a**) Under normal x-polarized incident waves. (**b**) Under normal y-polarized incident waves.

To validate the above-mentioned physical phenomena, we design a 1-D metasurface using ten optimal meta-atoms, with the overall dimension of 240 × 240 mm^2^. Full structure of the metasurface is full-wave simulated by the transient solver of CST Microwave Studio. The RCS of the metasurface and an equal-sized PEC surface with plane wave normally impinging are shown in Fig. 8. Significant reduction is obtained from 10–55 GHz for both polarizations. The metasurface can realize more than 7 dB RCS reduction in an ultra-wide frequency band from 11.3 to 51.3 GHz (up to a 4.54 octave bandwidth). The RCS reduction bandwidth is in good agreement with the possible bandwidth of manipulating EM waves predicted in Fig. 1(c). The new proposed mechanism based on geometric parameter optimization has been shown to greatly extend the bandwidth of RCS reduction.

**Figure 9:**
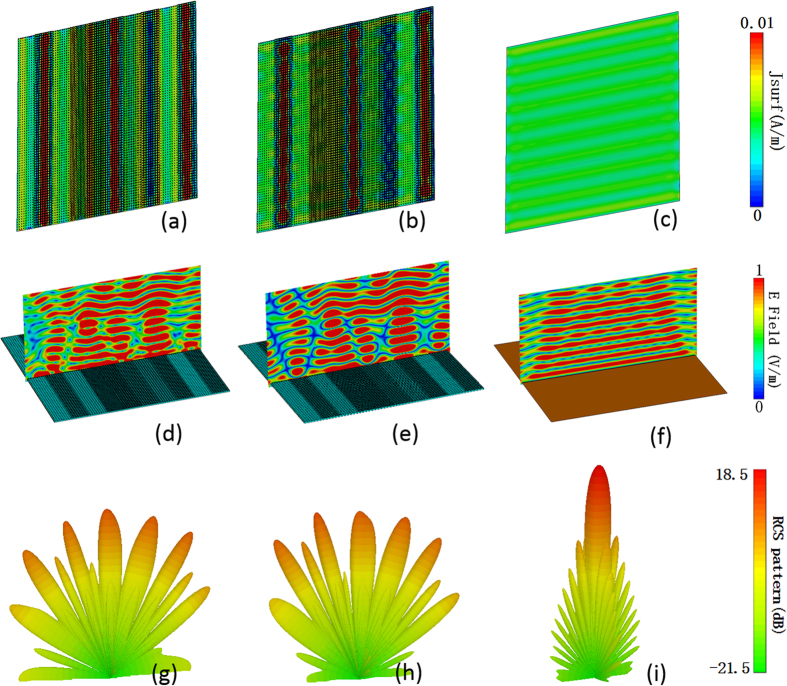
Simulated near-field and far-field patterns under x- and y-polarized normal incidence at 12 GHz. (**a**,**b**,**c**) The surface current distributions of the metasurface for x- and y-polarization and equal-sized PEC surface for x-polarization, respectively. (**d**,**e**,**f**) The near-field electric field distribution. (**g**,**h**,**i**) The far-field scattering patterns.

Figure 9(a–c) show the surface current distributions of proposed metasurface and the same-size PEC surface under normal incidence for both polarizations at 12 GHz. It demonstrates that the basic meta-atoms on the metasurface present different resonant states, which is critical to disturb the equiphase reflection (see Fig. 9(d–f)). As a result, the metasurface generates a diffusion scattering pattern in the far-field with suppressed amplitude as depicted in Fig. 9(g–i). Due to the non-uniform distributions of phase gradient between neighboring lattices, the specular reflections will no longer dominate within the whole scattered waves. The scattered energy is redirected to more directions and the scattered field is greatly suppressed at a very low level. Almost the same near and far field results (see Figs 8 and 9) for normal x- and y-polarized incident waves exhibit the polarization-insensitive feature of the proposed metasurface.

**Figure 10:**
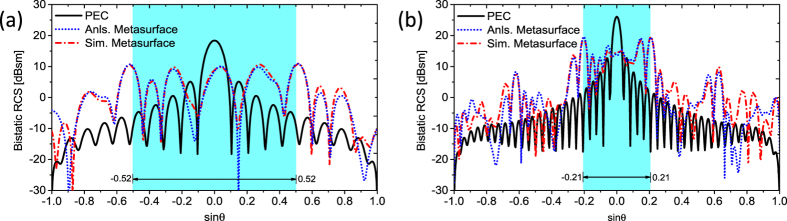
Comparison of the analytical and simulated results of far field scattering patterns. (**a**) At 12 GHz (**b**) At 30 GHz. Anls., Analyzation; Sim., Simulation.

Under normal incidence, the analytical and simulated results of RCS patterns along the principal planes (XoZ) at *f*_1_ = 12 GHz and *f*_2_ = 30 GHz are compared in Fig. 10. The corresponding periods of array factor are *P*_1_ = 1.04 and *P*_2_ = 0.42, respectively. Light blue area represents the main period. As shown, different diffusion scattering patterns are generated for these two frequencies due to different phase layout response. The scattering energy is concentrated in the main period and its vicinity due to the attenuation characteristic of element pattern (*EP*) as the scattering angle *θ* increases. Weak scattered field that appears away from the main period is caused by the side lobes of element pattern. Unlike the peak scattering of the reference PEC surface, flat scattering patterns are achieved by the metasurface. Comparisons of the analytical and simulated results of RCS pattern are demonstrated in Fig. 10. The analysis results are obtained by Eq. (1) using the simulated element pattern (*EP*). Good agreement confirms the validity of array pattern synthesis (APS) module, which provides a powerful mean for the fast and accurate analysis of metasurfaces with much less computing time and resources.

**Figure 11:**
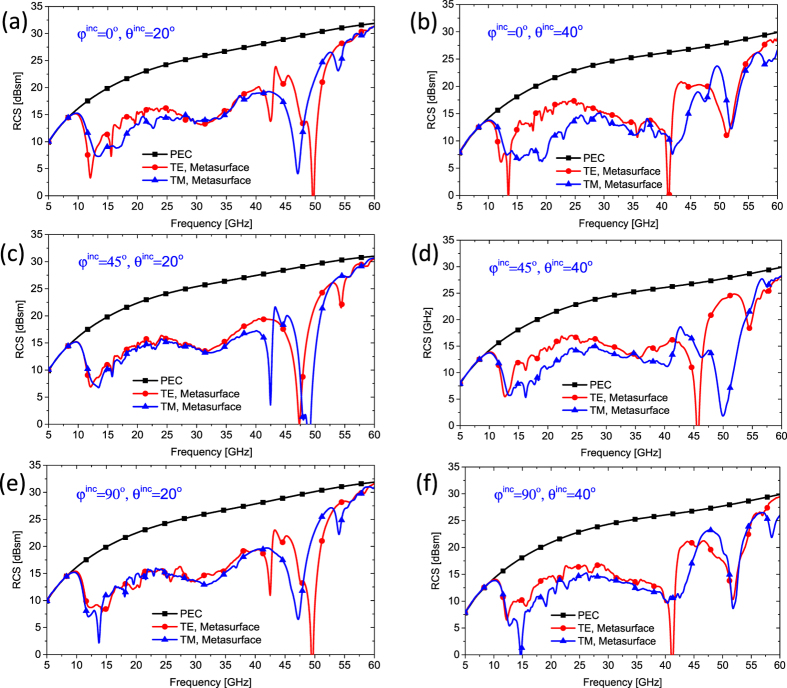
Simulation results of the metasurface under the oblique incidences. The RCSs in the specular reflection directions of the metasurface are simulated in frequency range from 5 to 60 GHz for both TE and TM polarizations. (**a**,**c**,**e**) Under 20° oblique incidence with azimuth angles of *φ*^*inc*^ = 0°, 45°, and 90°, respectively. (**b**,**d**,**f**) Under 40° wide angle incidence with azimuth angles of *φ*^*inc*^ = 0°, 45°, and 90°, respectively.

To investigate the incident direction dependence of the scattering profiles, two incident angles (*θ*^*inc*^ = 20°, 40°), three incidence planes (*φ*^*inc*^ = 0°, 45°, 90°) and both TE and TM polarizations are considered in our simulations. For the oblique incidence waves with different directions, the RCSs in the specular reflection directions based on Snell’s law are simulated. Figure 11(a–f) shows the simulated RCS of the metasurface across the ultra-wide frequency band (5–60 GHz), and exhibit excellent performance in suppressing the specular EM wave reflection. From these figures, we find the small scattering peaks when the observation angles equal to the incident angles between 10–55 GHz. Thus, for wide-angle incidence of arbitrary direction and polarization, the proposed metasurface still performs well in the working band.

**Figure 12:**
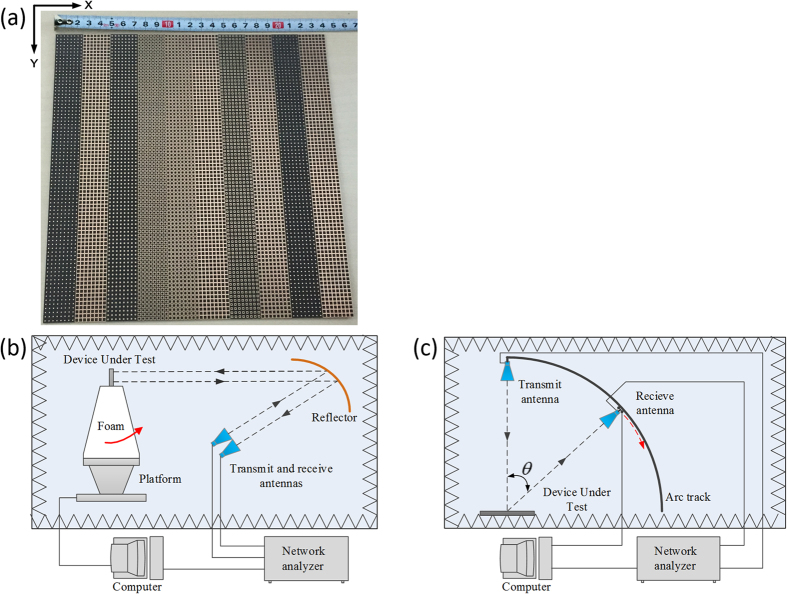
The measurement of the metasurface. (**a**) Photograph of the fabricated metasurface. (**b**) and (**c**) are schematic view of the monostatic and bistatic RCS measurement setup in two microwave anechoic chambers, respectively.

**Figure 13:**
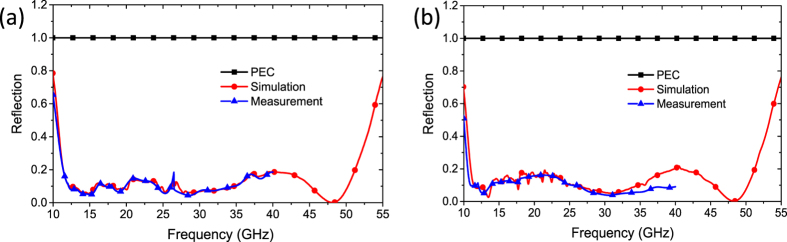
The simulated and measured backward scattering coefficients versus frequency. (**a**) Under normal x-polarized incident waves. (**b**) Under normal y-polarized incident waves. For the measured results in (**a**,**b**), the RCS of the metasurface and its metallic ground plane are separately measured first. Then, subtraction is made between their values.

To validate the predicted performance of our proposed metasurface, a sample is fabricated and its performance under normal incidence is measured. The sample is manufactured by LPKF ProtoLaser using printed circuit board (PCB) technology. The dielectric substrate is Rogers RT/duroid 5880 substrate with a thickness of 1.5748 mm and a dielectric constant *ε*_*r*_ = 2.2 (loss tangent tan *δ* = 0.0009). The metal patches and ground are 0.017 mm-thick copper layers. High-precision RCS measurement^34^ is conducted in the anechoic chamber of *Science and Technology on Electromagnetic Scattering Laboratory* in Beijing. Figure 12 shows the photo of the sample and the measurement setup. For monostatic measurement, two identical horn antennas are utilized as transmitting and receiving devices, respectively. Then, the scattering performance is evaluated by the transmission coefficients obtained by vector network analyzer. An equal-sized metallic ground of the proposed metasurface is also measured as reference. Figure 13 shows the simulated and measured monostatic RCS reductions (or reflection coefficients) versus frequency under normal incidence. The metasurface can achieve the simulated reflection coefficients less than 0.2 in an ultra-wide frequency band from 11.3 to 51.3 GHz for both polarizations. Due to the limitations of our experiment conditions, the measurement could not be conducted in the frequency range higher than 40 GHz. The measured reflection coefficients less than 0.2 is also achieved from 11.3 GHz to 40 GHz. Through the comparison of available results, we note that the measurements agree well with the simulations.

**Figure 14:**
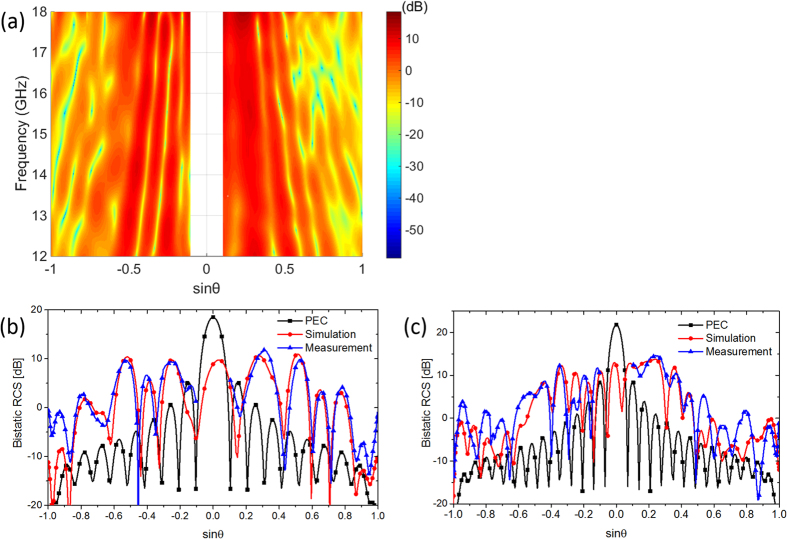
Measurement results of bistatic RCS for the metasurface. (**a**) Measured bistatic RCS along the principal (XoZ) plane in the frequency range from 12 to 18 GHz under normal incidence. (**b**,**c**) The simulated and measured bistatic RCS at 12 GHz and 18 GHz, respectively.

To verify the diffuse scattering characteristics of the metasurface, measurement of bistatic RCS under the normal incidence in a frequency band from 12 to 18 GHz is performed. For bistatic measurement, transmit antenna is fixed while receive antenna moves along an arc track to detect the reflection fields at scattering angle *θ* from 6° to 90°. Measurement results of bistatic RCS along the principal (XoZ) plane for the metasurface are presented in Fig. 14. The scattered energy is redirected to more directions, and the scattered fields are suppressed in a low level in all directions. The simulation and measurement results of bistatic RCS are also compared at 12 GHz and 18 GHz, respectively. As shown in Fig. 14(b and c), the change tendency is consistent with the simulation results. The value deviations can be attributed to measurement error resulting from the complex bistatic test system. Overall, the excellent performance of the proposed metasurface is confirmed.

## Conclusion

A one-dimensional metasurface based on size-adjustable meta-atoms is designed, fabricated, and tested for ultra-wideband specular reflection reduction. The best RCS reduction in an ultra-wide frequency band and the corresponding geometric parameters and arrangement of basic meta-atoms are obtained simultaneously by hybrid array pattern synthesis (APS) and particle swarm optimization (PSO) method. Intercepted EM energy is redirected to more directions and therefore the maximum of scattered field can be effectively suppressed at a low level based on energy conservation. Experiment results show good agreement with the corresponding simulation in terms of monostatic and bistatic RCS. The results show that the metasurface has a strong ability in suppressing specular EM wave reflection in an ultra-wide frequency band for both normal and wide-angle incidences. In addition, the bandwidth and amplitude of RCS reduction can be further extended by increasing the freedom degree in the selection of basic meta-atoms through the use of multiple shaped meta-atoms. Compared to previous approaches, our proposed metasurface for suppressing the specular electromagnetic wave reflection or radar cross section (RCS) has the advantage of easy fabrication, ultra-wide bandwidth and polarization independence of incident waves, which makes it promising for electromagnetic cloaking.

## Additional Information

**How to cite this article**: Su, J. *et al*. Ultra-wideband,Wide Angle and Polarization-insensitive Specular Reflection Reduction by Metasurface based on Parameter-adjustable Meta-Atoms. *Sci. Rep.* **7**, 42283; doi: 10.1038/srep42283 (2017).

**Publisher's note:** Springer Nature remains neutral with regard to jurisdictional claims in published maps and institutional affiliations.
